# Data describing Rax positive optic-vesicle generation from mouse embryonic stem cells *in vitro*

**DOI:** 10.1016/j.dib.2016.05.070

**Published:** 2016-06-03

**Authors:** Nozomu Takata, Mototsugu Eiraku, Eriko Sakakura

**Affiliations:** a Laboratory for in vitro Histogenesis, RIKEN Center for Developmental Biology, 2-2-3 Minatojima-minamimachi, Chuo-ku, Kobe, Hyogo 650-0047, Japan; b Center for Vascular and Developmental Biology, Feinberg Cardiovascular Research Institute, Northwestern University Feinberg School of Medicine, 303 East Superior Street, Chicago 60611, IL, USA

**Keywords:** Embryonic stem cell, Optic tissue, Rax, CHIR99021, GSK-3ß

## Abstract

This article contains data related to the research article entitled “Specification of embryonic stem cell-derived tissues into eye fields by Wnt signaling using rostral diencephalic tissue-inducing culture” Sakakura (2016) [1]. Mouse embryonic stem cells (ESC) were used for the generation of optic vesicle-like tissues *in vitro*. In this article we described data in which a Rax::GFP knock-in ESC line was used to monitor the formation of optic tissues. In addition, we also described the data of regional marker expression of Rax, Sox2 and Pax6 *in vivo* around the forebrain and the eye tissues for comparative purposes. These data can be valuable to researchers interested in investigating forebrain and eye tissue development.

**Specifications Table**

**Table:** 

Subject area	Biology
More specific subject area	Stem cell biology, Developmental biology, Regenerative medicine
Types of data	Image, graph, schematic diagram
How data was acquired	Inverted fluorescent microscope (fluorescent, bright-field) and Fluorescence-activated cell sorting (FACS) analysis
Data format	Raw, analyzed
Experimental factors	Mouse embryonic stem cells (ESCs) were differentiated into optic tissues *in vitro*
Experimental features	A chemical inhibitor, CHIR99021 (CHIR), which inhibits GSK-3ß, was applied for the generation of optic tissues in a three-dimensional manner using a chemically defined medium (CDM) and matrigel (MG).
Data source location	Laboratory for *in vitro* Histogenesis, RIKEN Center for Developmental Biology. Center for Vascular and Developmental Biology, Feinberg Cardiovascular Research Institute, Northwestern University Feinberg School of Medicine.
Data accessibility	Supplementary data of the article

**Value of the data**

• The expression pattern of Sox2, Pax6 and Rax provides the characterization of ESC-derived tissues for regional identification of neural tissues.
• The data and diagram for the timed-addition of reagents into the differentiation media may assist the readers in readily using the *in vitro* system for inducing optic tissues from ESCs.
• A data of the ESC-derived Rax positive tissues from culture day 4 to day 24 may give better information of the ESC differentiation culture for researchers in the related fields.

## 1 Data

**Fig. 1:**
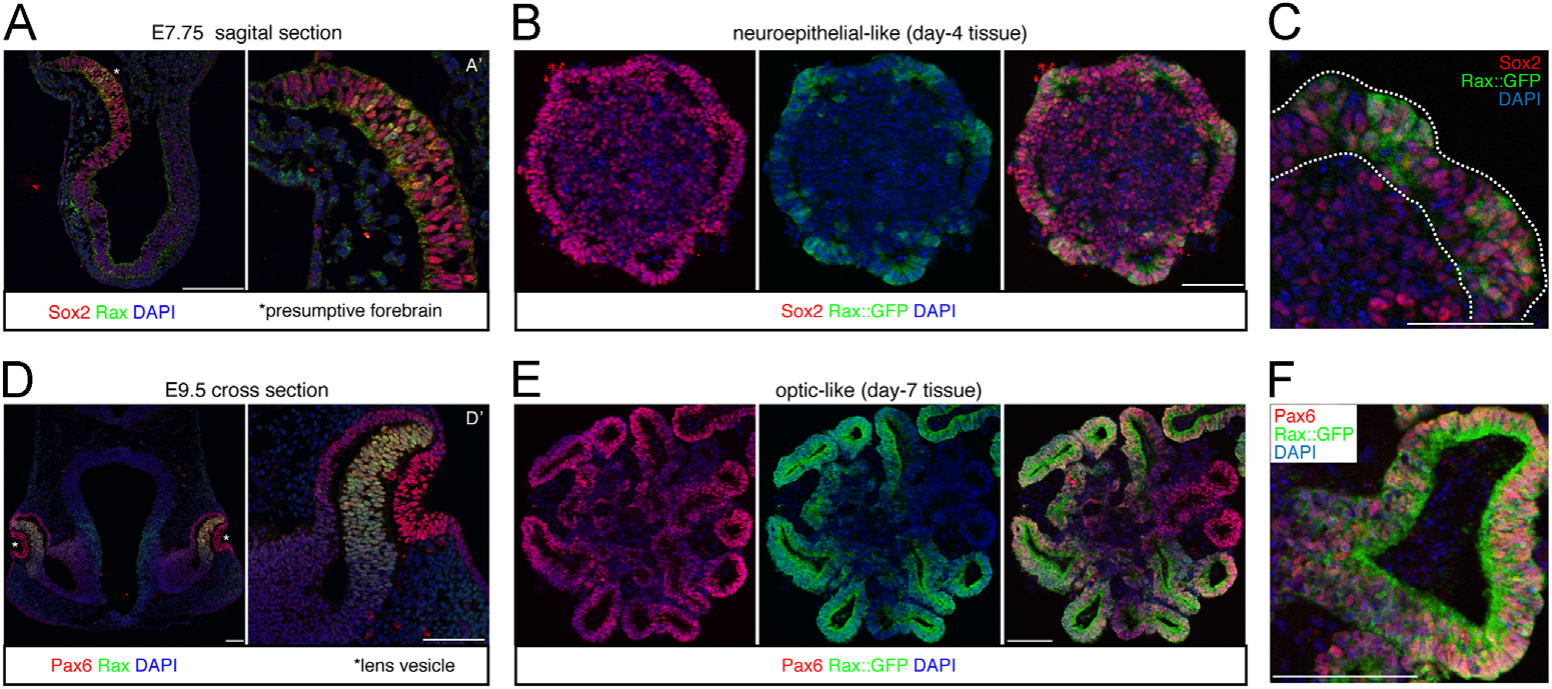
Comparison between *in vivo* and *in vitro* marker gene expression in the neuroepithelium and optic tissues. (A, B, C) E7.75 embryo and day-4 ESC-derived tissues show Sox2, Rax and Rax::GFP signals via immunostaining. Image B and C are prior to CHIR addition. Image A′ is a high magnification of the forebrain region in Image A. Dotted lines in image C indicate epithelial-like structures. (D, E, F) E9.5 embryo and day-7 ESC-derived tissues in CDM/MG/CHIR condition show Pax6, Rax and Rax::GFP signals via immunostaining. Image D’ is a high magnification of the eye region in image D. Scale bars; 100 µm.

**Fig. 2:**
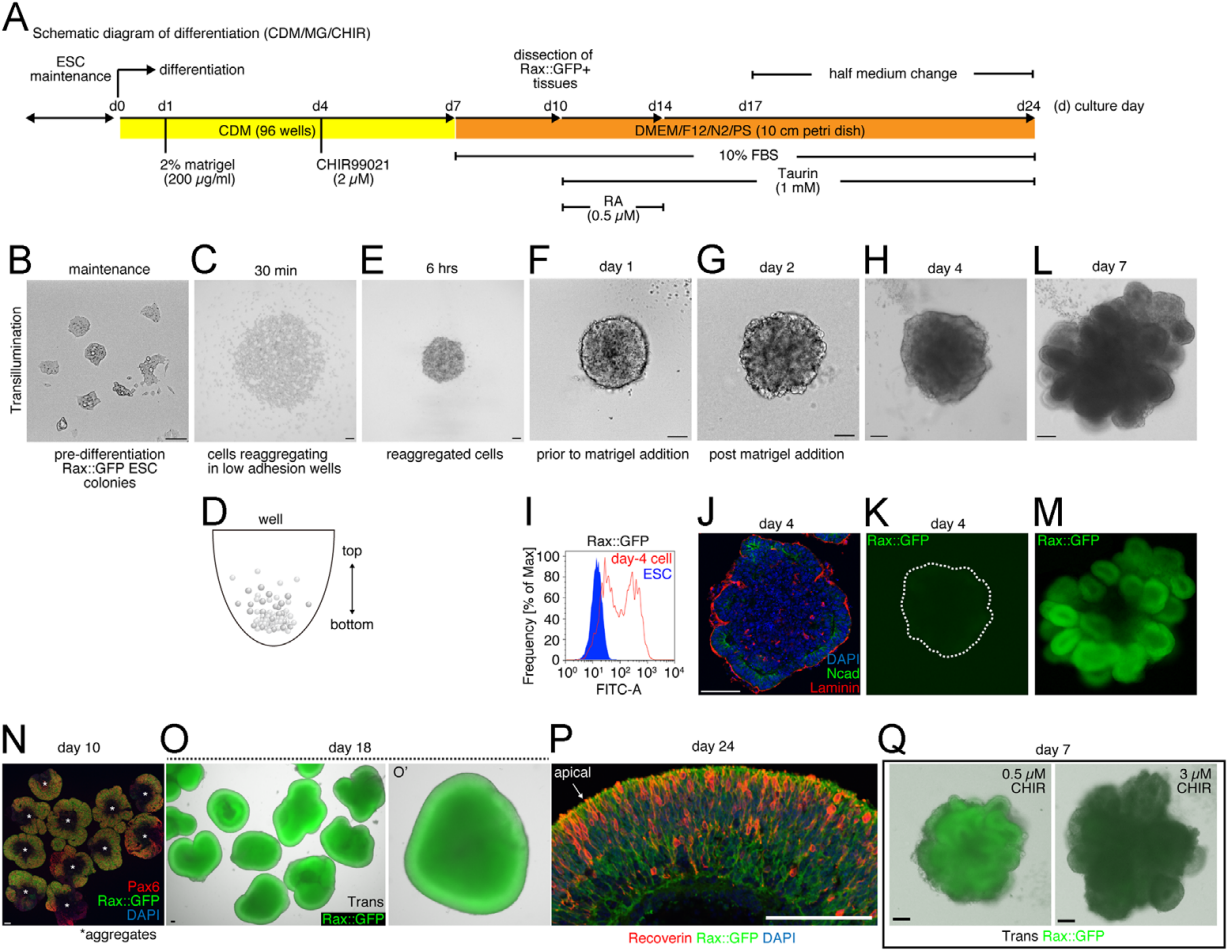
Schematic diagrams and data for the generation of optic tissues from mouse ESCs *in vitro*. (A) A brief step by step instruction in the CDM/MG/CHIR condition. (B, C, E–H, K–M, O, O′, Q) Transillumination and fluorescent images of living cells and tissues. Trans, transillumination. Images B, C, E-G were acquired via EVOS microscope. (D) Schematic of the quick reaggregation of ESCs. (I) FACS analysis of ESC-derived day-4 cell. ESC serves as a negative control. (J, N, P). Immunostaining of ESC-derived tissues showing N-cadherin (Ncad), Laminin, Pax6, Rax::GFP and Recoverin. Day-4 data are prior to CHIR addition. CDM, chemically defined medium. MG, matrigel. CHIR, CHIR99021. PS, Penicillin-Streptomycin. RA, Retinoic acid. Scale bars; 100 µm.

This data mainly focuses on describing the regional marker expression of neural tissues and the data of eye tissue-inducing culture (Fig. 1, Fig. 2), and refers to our recently published [1]. We used mouse embryo and mouse ESC-derived tissue samples to analyze the regional marker expression via immunostaining and also showed the images of ESC-derived tissues in living condition. The data shown are microscopy images (Fluorescent and Bright-field), graphs (Population of GFP+cells) and schematic diagrams (Step-by-step processes).

## 2 Experimental design, materials and methods

### 2.1 Regional marker expressions of neuroepithelium *in vivo* and *in vitro*

Rax was expressed in the anterior neuroepithelium, which expressed Sox2 as well at embryonic day (E) 7.75 (Fig. 1A, A′) [2]., [3]. The optic tissues at E9.5 co-expressed Rax and Pax6 (Fig. 1D, D′) [4]. Using a Rax::GFP ESC line, Rax+ tissues were locally induced at culture day 4, at which point Sox2 was globally expressed in the neuroepithelial-like structure (Fig. 1B, C). Subsequently, by adding CHIR99021, Rax+/Pax6+ optic vesicle-like structures were formed at culture day 7, reminiscent of *in vivo* optic tissues (Fig. 1E, F).

### 2.2 Step-by-step data during generating Rax+ optic vesicle-like tissues in the CDM/MG/CHIR condition

From here we describe data for generating Rax+ optic vesicle-like tissues using mouse ESCs (Fig. 2A). ESCs were maintained in maintenance medium before differentiation [5](Fig. 2A, B). At day 0, we performed SFEBq (Serum-free Floating culture of Embryoid Body-like aggregates with quick reaggregation) [6]. ESCs were dissociated and quickly aggregated in the non-adhesion coated 96 wells (Fig. 2D). 30 min later, we could observe the beginning of aggregation and, 6 h later, cells had mostly aggregated (Fig. 2C, E). At day 1, 2% matrigel was directly added into the medium (Fig. 2F). At day 2, the surface portion of the day-2 aggregate was changed morphologically (Fig. 2G). By day 4, neurorpithelial-like structures expressing apico-basal polarity marker, N-cadherin (inside), and Laminin (outside) were observed (Fig. 2H, J). Simultaneously, Rax::GFP cells started to faintly appear in the epithelial-like structure (Fig. 2I, K). This time (day 4), 2 µM CHIIR99021 was added, which inhibited GSK-3ß. At day 7, Rax+ optic vesicle-like structures were generated (Fig. 2L, M). Subsequently, at day 7, we replaced CDM with DMEM/F12/N2/10% FBS (fetal bovine serum) medium to grow tissues for long-term culture (Fig. 2A). At day 10, we chopped Rax+ tissue portions which also expressed Pax6 in order to separate them from other tissues (Fig. 2N). From day 10, we precisely followed the method reported previously [5]. We confirmed that day-18 tissues mostly expressed Rax::GFP (Fig. 2O, O′) and day-24 tissues expressed Rax::GFP and Recoverin, which is expressed in the photoreceptor cells of the eyes *in vivo* [7], [8] (Fig. 2P). The addition of 0.5 or 3 µM CHIR99021 from day 4 to day 7 did not efficiently: induce the formation of Rax+ vesicle-like structure (Fig. 2Q).

### 2.3 ESC culture and reagents

ES cell maintenance; Using ESC-qualified Fetal calf serum (FCS) and KnockOut Serum Replacement, a Rax::GFP knock-in ESC line was maintained as described previously [5]. ES cell differentiation; Based on SFEBq using Nunclon Sphera 96U Bottom Plate (Thermo Scientific, 174925) [6], the differentiation method was performed in the CDM/MG/CHIR condition for inducing Rax+ optic vesicle-like tissues. CDM was prepared as follows: IMDM, GlutaMAX™ Supplement (Thermo Fisher Scientific, 31980030)/ Ham׳s F-12 Nutrient Mix, GlutaMAX™ Supplement (Thermo Fisher Scientific, 31765035) 1:1, 1x Chemically Defined Lipid Concentrate (Thermo Fisher Scientific, 11905031), 450 µM 1-Thioglycerol (SIGMA-ALDRICH, M6145), 5 mg/ml Bovine Serum Albumin (SIGMA-ALDRICH, A3156) and 15 µg/ml apo-Transferrin bovine (SIGMA-ALDRICH, T1428). At day 0, ESC were dissociated into single cells and the number of cells was counted via trypan-blue staining (SIGMA-ALDRICH, T8154 for excluding dead cells) to seed 3000 cells per well in CDM. At day 1, 2% (vol/vol) of matrigel, growth factor reduced (BD, 354230) were added. The final protein concentration of matrigel is typically 200 µg/ml (from around 10 mg/ml protein concentration) and several matrigel lots are routinely tested using a previous eye inducing protocol [5]. At day 4, 2 µM CHIR99021 (STEMGENT, 04-0004) was added. Infrequently, ESC-derived tissue at day 4 does not expressed Rax::GFP (typically due to ESC-passage number or incorrect ESC maintenance state). In this case, the addition of CHIR99021 led to the formation of Rax negative tissues at day 7 (data not shown). At day 7, ESC-derived tissues were transferred into DMEM/F12/N2/Penicillin-Streptomycin (PS), containing 10% FBS (vol/vol). At day 10, from here, ESC-derived tissue were grown exactly the same as previously published [5], and its process is also briefly shown in Fig. 2A.

### 2.4 Immunostaining and acquiring images

Slc:ICR mouse embryos were purchased from Japan SLC, Inc. to serve as a positive control. Immunostaining of cryosectioned samples was performed as previously described [9]. Primary antibodies were used as follows: GFP (rat, 1/500, NACALAI, 04404-84), Sox2 (goat, 1/250, santa cruz, sc-17320), Rax (guinea pig, 1/1000, TaKaRa custum, MS8407-3), Pax6 (mouse, 1/500, R&D, MAB1260), N-cadherin (mouse, 1/1000, BD, 610920), Laminin (rat, 1/1000, CHEMICON, MAB1905) and Recoverin (rabbit, 1/1000, chemicon, AB5585). DAPI was used for counter staining. Immunofluorescent images were taken by LSM710 or 780 (Zeiss). For the visualization of living ESCs and ESC-derived tissues, KEYENCE (KEYENCE) and EVOS (Thermo Fisher) microscopes were used. Acquired images were merged using Photoshop and ImageJ softwares.

### 2.5 Fluorescence-activated cell sorting (FACS) analysis

FACS analysis was performed as previously described [9].
